# Service users’ perspectives on acute, outreach and outpatient psychiatric crisis interventions with and without peer support: results of a qualitative study

**DOI:** 10.1186/s12913-026-15319-y

**Published:** 2026-08-11

**Authors:** Lena-Katharina Oeltjen, Georg Knigge, Maike Schulz, Imke Heuer, Timm Neeland, Jasmina Schmidt, Claudia Römer, Kay Bultmann, Jörg Utschakowski, Candelaria Mahlke, Ansgar Gerhardus

**Affiliations:** 1 Department for Health Services Research, Institute for Public Health and Nursing Research, Health Sciences Bremen, University of Bremen, Bremen, Germany; 2 Department of Psychiatry and Psychotherapy, Hospital Bremen-Ost, Gesundheit Nord Klinikverbund Bremen gGmbH (GeNo), Bremen, Germany; 3 Clinic for Psychiatry and Psychotherapy, University Hospital Eppendorf, University of Hamburg, Hamburg, Germany; 4 Ministry of Health, Women and Consumer Protection, Department of Mental Health and Addiction, Bremen, Germany; 5 Public Health Department Bremen, Bremen, Germany

**Keywords:** Psychiatric crisis intervention, Peer support, Lived experience, Police involvement, Coercion, Qualitative research

## Abstract

**Background:**

Crisis intervention teams assist individuals in acute mental health crises. If unavailable or situations escalate, the police may be involved, and compulsory admissions may be initiated in case of risk to self or others. – measures often perceived as stigmatizing and traumatizing. Respectful, empathetic interactions can prevent escalation and reduce coercion. Peer support, by individuals with lived experience, can enhance recovery, improve quality of life, and reduce coercive measures. In the city of Bremen, the Crisis Intervention Service (CIS) handles emergencies via mobile teams or outpatient consultations. The “PeerIntervent” project integrated trained peer support workers (PSW) into two regional Crisis Intervention Service (CIS) teams. This study examines how individuals with severe mental health conditions experience care during acute, outreach and outpatient psychiatric crises.

**Methods:**

Semi-structured interviews were conducted with 25 individuals recently affected by psychiatric crisis interventions and analysed using qualitative content analysis.

**Results:**

CIS was involved in all cases; PSW in six cases, police in eight. Empathic listening, calm communication, transparent explanations, and involvement in decision-making were described as supportive. Confrontational communication or insufficient information was experienced as distressing. Structural factors, including staff continuity and the waiting environment, also shaped overall experiences. Peer support was largely perceived positively, with the sharing of lived experience associated with authenticity and hope. Experiences with police were described ambivalently, ranging from reassuring and supportive to intrusive and escalating.

**Conclusion:**

Findings highlight the importance of calm, empathic, and transparent communication for trust and perceived safety, supporting the need to strengthen targeted training for all crisis care providers, including police. In line with prior research, PSW may complement crisis care by providing experiential credibility and hope, but their use should remain context-sensitive and requires further study.

**Trial registration:**

The study was approved by the Ethics Committee of the University of Bremen on June 20, 2022 (reference: 2022-09) and was registered in the German Clinical Trials Register (DRKS) on July 4, 2022 (DRKS00029377).

**Supplementary Information:**

The online version contains supplementary material available at 10.1186/s12913-026-15319-y.

## Background

Mental health crises pose a significant challenge for those affected, their relatives, and society. In such situations, individuals experiencing acute distress or danger encounter professional support systems. In many countries, specialized crisis services take on the task of providing short-term assistance in acute emergencies, acting to de-escalate tensions and initiating further steps in care [1, 2]. If these services are unavailable or a situation threatens to escalate, the police are often additionally called in. If the crisis cannot be defused through dialogue and de-escalation, compulsory admission is permitted in Germany. As a last resort, such measures may be imposed under relevant state laws govering assistance and protective measures for individuals with mental illness [e.g. 3] and under the German Civil Code [4]. While these legal provisions provide a framework for intervention, they also raise serious ethical and personal concerns, as the use of coercion can profoundly impact those subjected to it and can constitute violations of fundamental rights. From the perspective of those affected, coercive measures are often experienced as discriminatory, stigmatizing, and traumatizing [5, 6]. Furthermore, experiences of coercion are frequently associated with feelings of fear, humiliation, and helplessness [7] and can reduce the likelihood of seeking help in future crises [8].

There has been a noticeable increase in compulsory admissions in many countries over the past years [9]. This trend is also evident in Germany and in the town of Bremen [10, 11]. Many professionals consider the use of coercive measures (such as compulsory admissions) to be inevitable in hindsight [12]. However, experiences reported by those affected suggest that such might be avoided more frequently if the quality of interactions were improved [13]. Individuals who have experienced coercive measures in inpatient settings report that alternative approaches could have been taken, such as improved communication, engaging with the patient on eye level, involving trusted persons, and considering the patient’s needs [7, 13]. Respectful, transparent, and empathetic communication, as well as the early involvement of individuals in decision-making, are described by those affected in inpatient care settings as de-escalating, trust-building, and potentially reducing the likelihood of coercive measures [14–16].

Although derived primarily from inpatient contexts, these observations suggest that psychiatric professionals’ interactions with patients can critically influence whether crises escalate [14]. Whether comparable dynamics apply to outreach and outpatient crisis settings — which differ in terms of environment, time pressure, and professional roles — remains largely unexplored. Building on this, psychiatry is increasingly adopting person-centered, recovery- and empowerment-oriented approaches [17, 18]. Within this framework, Peer Support Workers (PSW)—individuals with lived experience who support others during crises—offer a practical application of these principles. As part of the psychosocial care system, peer support enables people with personal experience of crises to assist others on their path to stabilization and recovery [19]. The underlying premise is that shared experiences foster trust, convey hope, and strengthen personal resources [20, 21].

In the city of Bremen, the Crisis Intervention Service (CIS) as part of the Public Health Service, is responsible for handling psychiatric emergencies. It operates on weekdays from 8:30 a.m. to 3:00 p.m. across five areas. At all other times, including weekends and public holidays, a central crisis service or the police is responsible. The CIS can be contacted in two ways. First, it is typically reached by phone—by the individual in crisis, the police, or a third party (relatives, citizens). The CIS assesses the urgency and, if necessary, sends a team of at least two staff members to visit the individual in crisis. If needed, the CIS requests police support to forcefully access the individuals’s home—for instance, to rule out self-endangerment or endangerment of others, or if immediate danger to service workers is expected based on prior experience. In some cases, the police arrive first and request CIS support instead. Second, the CIS offers open consultation hours, allowing individuals to visit without an appointment. In such instances, the crisis conversation takes place on-site (outpatient crisis). Compulsory admission may occur during such consultations, though this is rare [22].

The “PeerIntervent” project, an exploratory, cluster-randomized controlled trial (cluster-RCT) funded by the Federal Ministry of Health, integrated PSW into two of five CIS teams in the town of Bremen for one year to actively support crisis interventions and improve care. The two CIS teams with integrated PSW served as the intervention clusters, while the remaining three teams functioned as control [22]. The PSW involved in the project were trained through the specialized, europe-wide recognized Experienced-Involvement (EX-IN) program. This includes 250 h of instruction across twelve modules on topics such as recovery, trialogue (a collaborative exchange between service users, relatives, and professionals), and crisis intervention, as well as two practical phases of 120 h each [23].

While empirical evidence exists on how individuals experiencing psychiatric crises perceive inpatient care, much less is known about their experiences during acute, outreach and outpatient interventions. Existing research has identified factors that facilitate supportive relationships in crisis situations, but these insights are largely based on professionals’ perspectives [24]. However, the benefit of support strongly depends on how it is subjectively perceived by those receiving it [25]. Although the service user’s perspective has been explored in a small number of studies, there is still little evidence on the involvement of PSW, especially in outreach crisis settings [26]. The present study constitutes the qualitative component of the broader PeerIntervent trial and specifically addresses service users’ subjective experiences of care — a dimension not captured by the trial’s quantitative outcome measures. The present qualitative study therefore addressed the following research question: How do individuals with severe mental health conditions experience the provision of care during acute, outreach and outpatient psychiatric crises?

## Methods

### Study design

Semi-structured interviews were conducted with people who had experienced acute psychiatric crisis interventions. In addition, a short questionnaire was used to collect sociodemographic data to describe the sample. The study was approved by the Ethics Committee of the University of Bremen on June 20, 2022 (reference: 2022-09) and was registered in the German Clinical Trials Register (DRKS) on July 4, 2022 (DRKS00029377). All research activities were conducted in accordance with the Declaration of Helsinki. The reporting of this publication follows the COREQ checklist for qualitative research [27].

### Patients and recruitment

We aimed at including 15 individuals from the intervention and 15 from the control clusters who had experienced psychiatric crisis interventions to ensure a diverse range of perspectives and to support the development of meaningful thematic insights. Interviews were continued until thematic sufficiency was reached, meaning that the material provided sufficient depth and breadth to meaningfully address the research question. At the same time, we were aware that recruiting participants from this group can be challenging, and, given the heterogeneity of the group full saturation within each subgroup was not to be expected. Participants were eligible for inclusion if they were of legal age (18 years or older), had experienced a psychiatric crisis intervention approximately three months prior to recruitment and spoke either German or English. The crisis had to involve the CIS, while the involvement of police and/or peer support was optional. To achieve this, all individuals with a documented crisis event during the study period were contacted via a written postal invitation. The invitation was sent by a staff member from Gesundheit Nord (GeNo), the public network of hospitals in Bremen and part of the Public Health Service, who is responsible for organizing the CIS. Those interested in participating could return a stamped and pre-filled consent form to the research team. The research team then contacted the interested individuals, assessed their eligibility, and scheduled interview appointments. The interviews were conducted from October 2023 to May 2024.

### Data collection and procedure

The semi-structured interview guide was developed on the basis of the research question and in consultation with the trialogical advisory board (EmPeeRie NoW) of the Department of Psychiatry and Psychotherapy at the University Medical Centre Hamburg-Eppendorf EmPeeRie NoW comprises individuals with lived experience of mental crises as well as family members, thereby representing a broad range of experiential expertise [28]. A pretest was conducted with individuals who had prior experience as service users in psychiatric crisis interventions. After the pretest minor adjustments were made to the guide. The final version of the interview guide consisted of four main questions addressing the initiation and course of the crisis intervention, the progression after the acute phase, and further crisis experiences. Each main question was accompanied by supplementary sub-questions, which were only asked if the participant had not already addressed the relevant aspects on their own. Two slightly different versions of the interview guide existed, depending on whether a PSW was present during the crisis intervention or not. If a PSW had been involved, additional sub-questions were asked regarding the participant’s perception of the PSW behaviour during the crisis intervention. If no PSW had been involved, an additional two-part main question was included: first, the participant was asked whether they knew what a PSW is. If not, a brief explanation of the concept of peer support was provided. Afterwards, the participant was invited to reflect on how the course of the crisis might have unfolded if a PSW had been present.

To allow interviews to be conducted in English, the guide was translated and internally reviewed for clarity and accuracy. The interview guide can be found in the supplementary materials.

Prior to the interviews, all participants received detailed written and verbal information about the study objectives and participation requirements, including their right to withdraw from the study at any time without providing reasons and without incurring any disadvantages, as well as assurances of confidentiality, anonymisation of their data, and secure data handling in accordance with applicable data protection regulations. Written informed consent was obtained from all participants. Participants were allowed to bring a companion for support during the interview. Two participants selected this option. All interviews were conducted in person at the respective CIS where the participant was receiving services. This setting was chosen to ensure access to psychotherapeutic support if needed after the interview. Each participant was interviewed once. No repeat interviews were conducted. Interviews were carried out by LKO, a female researcher with a Master’s degree in Public Health and currently a PhD candidate in Health Services Research. Interviews were scheduled to last between 30 and 60 min. Participants received an allowance of 20 euros for their participation. Interviews were audio-recorded and subsequently transcribed verbatim. After each interview, field notes were taken to document the interview process. Transcripts were imported into MAXQDA (version 2020–15) for qualitative analysis.

### Data analysis

The material was analysed through a multi-stage process following the structuring qualitative content analysis according to Kuckartz [29]. Initially, two authors (LKO, GK) reviewed a selection of the interviews and generated case summaries and memos. They then deductively developed main categories based on the interview guides and memos and applied these categories to the selected material. In a subsequent step, additional main categories and all subcategories were inductively derived in collaboration with a peer researcher (CR) and applied to the selected data segments. Details of the coding framework can be obtained from the authors upon request.

The complete dataset was then coded using the refined category system. Due to the volume of material, coding was shared among four researchers (LKO, GK, JS, TN), who were organized into two pairs. Within each pair, both researchers independently coded half of the interviews. The results were then compared, and discrepancies were resolved through consensus.

Based on the fully analysed material, relevant categories were revisited and reanalysed with regard to the research question addressed in the present article. This analytical step was conducted by Author 1 (LKO) and validated by Author11 (AG). To avoid placing additional burden on participants, transcripts and derived findings were not returned to participants for feedback.

Sociodemographic data from the questionnaires were analysed descriptively.

### Research team

The authors have academic backgrounds in Public Health (LKO, AG, TN), Psychology (GK, CM, JS), Sociology (MS), and Literary and Cultural Studies (IH). All authors possess extensive experience in qualitative research. Their interest in the topic emerged from their work on peer support and psychiatric care. The authors had no personal relationships with the interview participants beyond the conduction of the interviews. Participants were informed about the authors’ academic roles and the purpose of the study, but not about any personal interests of the authors. Throughout the conduct and analysis of the interviews, the authors made a conscious effort to maintain a neutral stance. Reflexivity was supported through the writing of memos after each interview, regular team discussions to negotiate interpretations, and collaborative category development involving a peer researcher with lived experience of mental health crises. Authors remained aware of possible personal preconceptions during both data collection and data analysis.

## Results

### Sample characteristics

A total of 27 individuals were interviewed. Two individuals were excluded as they had not experienced an acute crisis intervention according to study criteria, leaving 25 for analysis. Of the 25 interviews, 14 were conducted with participants from the control group and 11 with participants from the intervention group (see Table 1). All crisis interventions involved a CIS team; in six cases only the police were additionally present, in four cases only a PSW accompanied the CIS, and in two additional cases both a PSW and the police were present. Eleven interviews involved patients from outreach crisis interventions, while 14 involved outpatients. Five participants reported experiences of coercion, including compulsory admission and informal coercion (e.g. perceived pressure to cooperate, with the possibility of coercive measures in case of non-compliance) in the context of crisis interventions.

**Table 1:** Professionals involved in the crisis intervention

Professionals involved in crisis situations alongside those affected	Total *N*	Intervention Group *N*	Control Group *N*	Outreach *N*	Outpatient *N*
CIS	13	3	10	2	11
CIS and Police	6	2	4	6	-
CIS and PSW	4	4	-	1	3
CIS, Police and PSW	2	2	-	2	-
**Total**	**25**	**11**	**14**	**11**	**14**
*Crisis Interventions involving CIS*	*25*	*11*	*14*	*11*	*14*
*Crisis Interventions involving Police*	*8*	*4*	*4*	*6*	*-*
*Crisis Interventions involving PSW*	*6*	*6*	*-*	*3*	*3*

Participants’ genders were relatively evenly distributed (see Table 2). 6 participants were aged 18–30 years, 4 were 31–40 years, 3 were 41–50 years, 8 were 51–60 years, and 4 were over 61 years old. Most Participants held German citizenship (22). The majority (19) reported both parents being of German nationality. Participants’ educational qualifications were categorized according to International Standard Classification of Education (ISCED) levels [30]: Overall, 20 participants were classified at ISCED level 3 (upper secondary education). 2 participants had completed lower secondary education (ISCED 2), 2 reported other types of school-leaving qualifications that could not be clearly assigned to a specific ISCED level, and 1 participant had left school without obtaining a formal qualification (below ISCED 2). Employment status included 8 full-time, 4 part-time, 3 marginal employment, and 10 unemployed/retired. The majority of participants were unmarried (13). Household size ranged from 1 to 4 people, with 13 participants living alone.

**Table 2:** Sociodemographic characteristics of participants (*N* = 25)

Characteristic	*n* (%)
**Gender**	
Male	12 (48.0)
Female	13 (52.0)
**Age (years)**	
18–30	6 (24.0)
31–40	4 (16.0)
41–50	3 (12.0)
51–60	8 (32.0)
≥ 61	4 (16.0)
**Parental Nationality**	
Both parents German	19 (76.0)
One parent German	1 (4.0)
Neither parent German	5 (20.0)
**Educational Qualification (ISCED)**	
Below ISCED 2 (no qualification)	1 (4.0)
ISCED 2 (lower secondary)	2 (8.0)
ISCED 3 – university entrance qualification	14 (56.0)
ISCED 3 – intermediate secondary certificate	6 (24.0)
Other/unclassifiable qualification	2 (8.0)
**Employment Status**	
Full-time	8 (32.0)
Part-time	4 (16.0)
Marginal employment	3 (12.0)
Unemployed/retired	10 (40.0)
**Marital Status**	
Unmarried	13 (52.0)
Divorced	6 (24.0)
Married	3 (12.0)
Widowed	2 (8.0)
Married but separated	1 (4.0)
**Household**	
Living alone	13 (52.0)
Household size 2–4 persons	12 (48.0)

### Interview characteristics and codebook

The interviews lasted between 10 and 46 min (median = 22 min and mean = 22:45 min). Sixteen main categories were constructed from the data, with 55 subcategories emerging during the iterative analysis process. A total of 1368 codes were assigned across all interviews. Due to the large number of categories, overarching themes were developed to clarify and organize their presentation in this article. In line with the research question, the identified main and subcategories were systematically grouped under these themes. An overview of the overarching themes and their associated categories is presented in Table 3.

**Table 3:** Overview of overarching themes and associated categories

Overarching Theme	Associated main category	Associated sub category
1.) Reasons for crisis contact and initiation of contact	Events leading up to contact	Reason for crisis contact
	Initial contact	with other institutions *> with Police* *> with CIS*
2.) Perceived communicative, structural, and personnel influences on CIS crisis response	Expectations and feelings during contact	Feeling of being acknowledged
		Self-reported behaviour
		Contact evaluation *> neutral* *> negative* *> positive*
	CIS perception	Perceived CIS staff behaviour *> positive* *> negative*
		Expectations for contact with CIS
	Coercion experiences	Feelings of pressure and threat *> CIS*
	Wishes and suggestions for improvement	CIS
		Others
3.) Perceived decision-making responsibility in crisis context	Perceived decision-making responsibility	Shared, informed decision-making
		Paternalistic decision-making
4.) Post-crisis support	After the contact	Crisis debriefing > *None* *> With professionals* *> With relatives/friends*
	Wishes and suggestions for improvement	Others
5.) Peer support as a complementary form of assistance	PSW perception	Explanation of the PSW role by attendees
		Perceived meaningfulness and support from the peer
		Perceived PSW behaviour *> Description of behaviour* *> Perceived behaviour*
	Potential role and impact of a PSW in crises	Assessment of potential usefulness of peers in crises
		Knowledge and understanding of the peer support concept
	Wishes and suggestions for improvement	PSW
6.) Perceived behaviour of the police	Perceived police behaviour	Expectations for contact with CIS
		Perceived Police behaviour *> positive* *> negative*
	Coercion experiences	Retrospective evaluation of avoiding coercion
		Details of coercive measures
		Experience of coercion
		Feelings of pressure and threat *> Police*
	Wishes and suggestions for improvement	Police

### Content analysis

In the following, the results are presented according to the overarching themes and supported with illustrative quotes. Following a translation–back-translation procedure, the quotations were translated into English by Author 1 (LKO) (translation assisted by ChatGPT-5.3, OpenAI) and back-translated by an independent researcher to ensure the accuracy of the content.

#### Overarching theme 1: reasons for crisis contact and initiation of contact

In the outreach contacts, the affected individuals were predominantly in highly dynamic, sometimes chaotic crisis situations. They often reported escalating conflicts in their private environment, which in some cases led to physical altercations or self-harming behaviour, or exceptional situations such as psychoses, substance intoxications, or relapses following detoxification treatments. Initial contact was usually made through the police, who were generally alerted by family members or neighbours. During the interventions, the police frequently involved the CIS for additional support.

*I then climbed onto the balcony*,* onto the railing […] and threatened to jump down. […] I cried*,* I screamed. And one of the neighbours […] from across the street must have seen it [and called the police]. — BO3*,* Pos. 3*,* Outreach*.

*I was sitting in my room and had been drinking quite a lot of alcohol and also using cannabis. […] And I wanted to take my own life. […] I somehow managed to warn my girlfriend by writing somewhat cryptic messages. She knew that something was wrong. I then stopped responding to calls altogether. […] Then the police were called. — BO15*,* Pos. 3*,* Outreach*.

In contrast to outreach interventions, outpatient patients usually sought the counselling centres on their own initiative, directly during an acute internal crisis. The triggers were primarily highly distressing feelings such as anxiety, hopelessness, depressive tension, or suicidal thoughts, which were experienced as unbearable. The help-seeking process often developed following a period of increasing strain, sometimes also after unsuccessful attempts to find relief through other services; in some cases, a brief telephone contact with subsequent timely acute counselling preceded the personal visit.

*Yes*,* I was here so acutely because I was shocked*,* […] by myself*,* that it wasn’t just the confrontation with suicidal thoughts*,* but with suicidal thoughts of how I would actually carry it out. — BO6*,* Pos. 98*,* Outpatient*.

#### Overarching theme 2: perceived communicative, structural, and personnel influences on cis crisis response

##### Perceived communicative influences

There were different preferences in communication. On one hand, it was reported that clearly and firmly confronting the situation had been helpful,

*And of course I was then confronted firmly by the doctor*,* who said*,* severe depression in combination with poly-substance use*,* in combination with suicidal thoughts. […] That was also good*,* somehow*,* I come from a conservative and strict household and was also in the military and so on*,* and somehow making a clear statement like that was also good. — BO6*,* Pos. 101–103*,* about CIS*,* Outpatient*.

Others described experiencing this type of communication as too harsh:

*And on the other hand*,* the psychologist who was there said*,* very bluntly*,* you don’t die from a panic attack. I didn’t want to die either*,* but it didn’t exactly help me. — BM6*,* Pos. 16*,* about CIS*,* Outpatient*.

Participants described it as particularly positive when CIS staff explained the possible next steps and highlighted that it was possible to get back in touch afterward to ask for support.

*He said*,* I can call him anytime*,* he informed me about this crisis service*,* that I can turn to it if things really don’t work out*,* I could go to the emergency department*,* so he really gave me quite a lot to work with. — BO9*,* Pos. 28*,* about CIS*,* Outpatient*.

One participant described that they missed the information on the next steps:

*That someone would stand by me. That I would have someone who*,* calmly*,* without any protocols*,* first explained things to me and then supported me. — BO7*,* Pos. 24*,* about CIS*,* Outreach*.

Participants described that the calm manner of CIS staff helped them to open up. They experienced it as positive when CIS staff listened, asked questions, and allowed for pauses. This made the affected individuals feel acknowledged and gave them a sense of not being alone.

*[J]ust so calming*,* and also the way he asked the questions – he always knew exactly where to intervene to put the whole picture together properly*,* so that one […] loses inhibition and really talks freely […]. He also allowed pauses*,* […]*,* and repeatedly reassured me*,* ‘It’s all okay*,* you are not alone*,* we*,* we will find a way.’ — BO9*,* Pos. 15–16 + 19–22 about CIS*,* Outpatient*.

Behaviour by CIS that was perceived as empathetic and compassionate fostered a sense of being understood among the affected individuals.

*I remember that I was quite glad because they were very empathetic*,* I don’t know. […] It was somehow completely different*,* they weren’t like*,* it didn’t seem directed against me*,* and not like I had done something wrong*,* but rather*,* you know*,* they were understanding. — BO3*,* Pos. 12–13*,* about CIS*,* Outreach*.

Being allowed to speak without interruption, being listened to, and finding a compromise together were described as very positive and conveyed calmness as well as the feeling of not being alone.

*They gave examples*,* acknowledged me*,* and didn’t leave me alone*,* but we had a dialogue*,* an exchange*,* which then […] took away some of my fear so that I could ground myself again. Because I had a connection*,* a transfer*,* and that really supported me in that moment. […] I felt understood*,* simply accepted as I am in the moment. And I also experienced no judgment or anything like that; I’ve experienced it differently here before. — BM11*,* Pos. 11 + 17*,* about CIS*,* Outpatient*.

Acknowledging the situation as something that need not be a source of shame, and emphasizing that it is an illness for which the affected person is not to blame, was perceived very positively and fostered a sense of being acknowledged.

*[That] this crisis service […] also tried to make me understand […] that one does not have to be ashamed. ‘There’s no need for you to feel embarrassed about that.’— BO5*,* Pos. 13*,* about CIS*,* Outreach*.

Situations in which the affected individuals felt misunderstood were described as particularly negative.

*Unfortunately*,* I thought it was a complete disaster. […] I didn’t feel supported or acknowledged at all with my issues. I mean*,* I believe he did his best*,* but it was just completely the wrong person for the situation. — BO12*,* Pos. 4 + 26*,* about CIS*,* Outpatient*.

##### Perceived structural influences

The design of premises was only discussed by participants who had an outpatient crisis situation. Some participants described the waiting period before the outpatient crisis conversations as uncomfortable and stressful.

*The waiting area and the other patients waiting there really frightened me*,* and I felt very*,* very uncomfortable. — BM6*,* Pos. 12*,* about CIS*,* Outpatient*.

*It was hell for me. I was afraid the whole time that someone would see me. I felt completely left alone there. — BO12*,* Pos. 4*,* about CIS*,* Outpatient*.

In this context, participants also expressed the desire for the CIS counselling centre to provide a more welcoming environment where they could stay after the crisis conversation.

*Something that cushions you a bit more. Even if it’s just*,* here is a lounge*,* there are books*,* you can stay as long as you want or something like that. And I come by every hour and a half to check on you. Or something that gives you the feeling that you’re not completely alone. — BO12*,* Pos. 7*,* about CIS*,* Outpatient*.

##### Perceived personnel influences

Participants described how important it was to already know CIS staff or to be able to trust them. One participant reported that it had given them a good feeling to already know a staff member.

*And that made it*,* for me*,* yes*,* a little easier*,* that there was someone I knew*,* who*,* you know*,* also knows me. — BO5*,* Pos. 3*,* about CIS*,* Outreach*.

Another participant, in contrast, described feeling insufficiently supported by the staff. They had approached an CIS staff member while experiencing severe depression, but that staff member’s focus was on substance use disorders. They did not feel well supported.

*And the person seemed*,* honestly*,* not competent to me at all. […] At some point*,* he told me that he is responsible for addiction counselling. And basically the case I had was not his area at all. — BO12*,* Pos. 4*,* about CIS*,* Outpatient*.

For another participant in an outreach crisis situation, the involvement of an additional staff member during the course of the conversation was perceived as a turning point.

*And then the other person*,* the woman who was there – when the other one realized he just wasn’t getting through to me with those methods*,* the other one came over to me and was also very calm*,* sat down next to me. And then we first talked a bit about what the situation actually was*,* and that definitely helped me calm down a little again. — BO15*,* Pos. 31*,* about CIS*,* Outreach*.

A similar pattern was also observed in an outpatient crisis conversation. Initially, the affected person had the impression that the staff member did not want to help them; only when another staff member joined the conversation they felt acknowledged.

*That annoys me. This man does not want to help me. I feel that. He said I am a foreigner*,* I lie about everything. I find that strange. […] But the female doctor was very nice. — BO11*,* Pos. 16–19*,* about CIS*,* Outpatient*.

In one case where CIS staff approached the person in crisis as a group of three, this was described as overwhelming.

*I still felt a bit overwhelmed because there were three of them. — BM8*,* Pos. 3*,* about CIS*,* Outreach*.

#### Overarching theme 3: perceived decision-making responsibility in crisis context

The decision-making process was experienced as particularly positive by participants when they felt that they were actively involved in decisions about the next steps after the acute crisis and when different options were presented transparently. In particular, the opportunity to choose between different forms of care was perceived positively.

*And beforehand*,* the psychologist told me that they offer three types of services: the ward*,* the day clinic*,* and outpatient care*,* and [asked] what I would see as best suited for me. […] I then expressed again that I wanted the day clinic. […] Outpatient care was not enough for me*,* because I somehow needed to get out of my house. Being able to choose was just a dream. I have to say that. Really amazing. — BO6*,* Pos. 6 + 54*,* about CIS*,* Outpatient*.

In addition, one participant described that they was able to make their own decision despite the treatment team’s preferred alternative. Even when it became clear that the CIS team favoured a different approach, their decision was respected. This was experienced as an important sign of autonomy.

*I don’t think it was satisfactory for the two of them [from the CIS]. […] NAME kept saying again and again*,* ‘You can come with us […] we’re going now.’ And I thought*,* come on*,* leave me alone*,* right? And*,* yes*,* so I did feel seen […] So what I actually want to say is that I was satisfied with the staff here*,* and as I said also with the crisis service people*,* I felt seen. And well*,* if I say*,* ‘I don’t want to*,*’ I’m not being deprived of my rights. — BO5*,* Pos. 16–17*,* about CIS*,* Outreach*.

In other cases, decision-making was described as a collaborative process, in which the participant gradually arrived at a suitable decision through exchange with CIS staff. By contextualizing the situation and outlining possible consequences, an initially non-preferred inpatient stay could be accepted as a helpful option.

*And I had no energy left. I was really exhausted. […] And [the CIS staff were] helpful and told me*,* […] they would suggest that I go to the hospital for inpatient care. […] And then I said*,* okay*,* yes*,* they are right*,* that is a good way. — BM3*,* Pos. 3*,* about CIS*,* Outreach*.

Another participant noted that in the acute situation, it felt overwhelming to have to make decisions on her own to determine what was best for them. This highlighted a need for more guidance and clearer recommendations from the professionals.

*But otherwise he kept saying*,* well*,* you have to know what you need now. But that was a moment where I*,* I didn’t know what I needed. I would have wished that someone told me*,* like*,* this or… something*,* you know. And that was really terrible. — BO12*,* Pos. 4*,* about CIS*,* Outpatient*.

One participant reported that the decision for an inpatient admission was ultimately made without their active involvement. Although it remained unclear whether they had been formally asked for consent, they described that the decision was made over their head and that they eventually complied without resisting.

*And then*,* apparently*,* this solution was found somehow. I guess they then said that it was probably the best for me. […] Maybe they asked me*,* […] once more. […] But I think*,* I don’t remember. I think it could be that I once said ‘no’ or something like ‘nah’ or ‘yes.’ But I definitely know that when it was said*,* it’s going to the hospital now*,* […] I didn’t resist in any way. — BO14*,* Pos. 32–35*,* about CIS*,* Outreach*.

Another participant described a similar experience – consent to the admission was given less out of conviction and more due to feeling pressured and perceiving no alternatives.

*And it just kept getting more hectic*,* and I then knew*,* okay*,* if I don’t come along voluntarily now*,* it’s going to get very*,* very unpleasant. And then at some point I said*,* okay*,* yes*,* I’ve tried*,* I actually didn’t want to go*,* but yes*,* I’ll come along. — BO15*,* Pos. 31*,* about CIS*,* Outreach*.

One participant reported that during the acute crisis they was unable to make an independent decision. For this reason, they followed the CIS’s decision in order to feel safe.

*At that moment*,* I really didn’t know at all*,* I couldn’t make a healthy decision. — BO7*,* Pos. 16*,* about CIS*,* Outreach*.

#### Overarching theme 4: post-crisis support

One participant reported that the acute crisis was reviewed and processed in a follow-up conversation with a professional.

*Yes*,* so I had another conversation with him in between*,* and then we went through it again*,* he revisited exactly the points where we had left off. — BO9*,* Pos. 32*,* about CIS*,* Outpatient*.

The majority of participants reported that such a follow-up discussion did not take place, but in retrospect they would have wished for it.

*Well*,* but that’s something*,* I think*,* now that you mention it. And I’ve never thought about reviewing a crisis day like that*,* that would probably be a good idea. That’s*,* um*,* I hadn’t come to that question or idea at all yet. — BO5*,* Pos. 31*,* about CIS*,* Outreach*.

#### Overarching theme 5: peer support as a complementary form of assistance

In the following, findings are presented separately for participants who had a PSW present during their crisis intervention and those who reflected on the potential value of peer support without having had a PSW present during their crisis intervention.

##### Perceived support from PSW

Participants who had a PSW present during the crisis conversation in the outpatient setting described this as very helpful and supportive. In particular, the communication, perceived as empathic and sensitive, as well as speaking from a patient perspective, were highlighted positively.

*He was also very personal*,* spoke about himself*,* gave examples*,* met me where I was*,* and didn’t leave me alone with it. Instead*,* we had a dialogue*,* an exchange*,* which reduced my fear a little so that I could ground myself again. […] He did that really*,* really well—very empathic and sensitive. — BM11*,* Pos. 11*,* Outpatient with PSW*.

*But in the end*,* I think what really mattered was that he knows the patient perspective. — BM4*,* Pos. 15*,* Outpatient with PSW*.

In another example, participants described that the PSW was a very immediate and accessible contact person.

*The PSW actually spoke with me quite directly; he was actually the first one to talk to me like that. — BM4*,* Pos. 9*,* Outpatient with PSW*.

Participants also positively noted that the PSW role was explained from the very beginning.

*It was definitely pleasant to talk to him because I knew that he was a PSW. […] And it was also explained to me what it means that he is a recovery companion.* — *BM4*,* Pos. 9*,* Outpatient with PSW*.

In contrast, for participants who had a PSW present during an outreach crisis, it was not clear that the person was a PSW. The PSW stayed in the background, and the participants could not identify their role.

*I thought that […] they were three of them just to ensure their safety. That’s how it seemed to me. The man was really huge and broad. I thought he was just a security guard.* — *BM8*,* Pos. 14–15*,* Outreach with PSW*.

In retrospect, participants expressed the wish that the PSW could have interacted more actively during the outreach crises.

*He didn’t say anything himself*,* was very quiet*,* which was noticeable*,* and he kept nodding. So*,* I was right about everything. He just nodded and said yes*,* yes*,* yes*,* yes. I thought*,* okay*,* a critical question once in a while would have been nice. — BM1*,* Pos. 3*,* Outreach with PSW*.

*Yeah*,* maybe he could have introduced himself and said*,* ‘Hey*,* I’m a PSW or something like that. That would have been good if he had done that from the start. Because I don’t think he said anything at all. — BM8L*,* Pos. 21*,* Outreach with PSW*.

##### Potential support from PSW

The majority of participants who did not have a PSW present during the crisis stated that they could well imagine a PSW being helpful in their situation, particularly because a PSW could provide a feeling of not being alone and of having already experienced a similar situation themselves.

*So*,* I think it’s good because […] when you’ve experienced firsthand what these illnesses do to you*,* no one understands it as well as someone who has gone through the same thing […] that’s a really important sense of togetherness*,* you know*,* you’re not alone*,* we know how you feel*,* we really all know. […] And right away*,* you feel a connection*,* you immediately trust these people. — BO9*,* Pos. 39–40*,* Outpatient without PSW*.

The ability to understand and empathize with the affected person was also seen as a potentially major strength of a PSW.

*At least they know what it’s like. All those stupid psychologists*,* they only have theory*,* they don’t know what it’s like*,* and so on. […] And I could imagine that a PSW would have helped a lot. — BO3*,* Pos. 30–31*,* Outreach without PSW*.

Furthermore, it was explained that a PSW could provide a sense of security in situations where the affected person feels alone compared to the others present.

*But having someone sitting on my side. Because it was like*,* the CIS staff and I was sitting here*,* my parents were there*,* the officers were there. And it would have been good if someone had been next to me*,* so to speak. That would have been*,* yes*,* I think it would have felt good somehow*,* or a sense of security somehow. — BO14*,* Pos. 61*,* Outreach without PSW*.

Participants emphasized how important it is to relieve fear during a crisis situation. A PSW could do this particularly well by explaining what is happening and what will happen next, free from protocols and rigid structures.

*And that someone at least takes away a bit of the fear and says*,* this is how it is now*,* and this is how it will go*,* because you don’t know what’s going to happen. And I do think that would definitely be helpful. […] That someone stands by me. That I would have someone who calmly explains things without any protocols and then supports me. — BO7*,* Pos. 24 + 32*,* Outreach without PSW*.

Additionally, the potential role of PSW was described as both emotional and practical support. In particular, when other staff have limited time, a PSW was seen to be able to provide extra assistance for both the affected persons and the other professionals.

*That it’s simply a person who*,* because the other [CIS] staff was busy*,* it was really crowded downstairs*,* maybe just takes the time to have a cup of tea with you or go for a walk to the water or something. Yes*,* something like that. That would have helped me a lot*,* a feeling of*,* I’m not alone right now. — BO12*,* Pos. 60*,* Outpatient without PSW*.

Despite predominantly positive assessments, there were also uncertainties. Some participants expressed concern that a PSW might not yet be stable enough to support others.

*I would be afraid that the person isn’t ready yet to support me.* — *BO13*,* Pos. 35–39*,* Outpatient without PSW*.

A limited ability for PSW to act in crises was noted when affected persons were at risk of harming themselves or others, and coercive measures were necessary.

*Well*,* with psychoses*,* I don’t know how it is there*,* whether people might then*,* these are conditions I’m not familiar with. Maybe they are still a danger to themselves or others*,* and they say*,* we have to take them now. But I would say*,* with depression or addictions*,* addicted people […] there’s no danger. — BO5*,* Pos. 59*,* Outreach without PSW*.

#### Overarching theme 6: perceived behaviour of the police

Participants described the behaviour of the police as ambivalent.

In particular, showing understanding made participants feel acknowledged in their situation.

*And the police officers*,* there were two gentlemen*,* and they were super nice. They could completely understand my situation. — BM3*,* Pos. 3*,* about the Police*,* Outreach*.

One participant reported not feeling seen, as the experiences they described to the officers were dismissed as mere imagination.

*And at that moment*,* when I was already surrounded by five or six police officers […]*,* he then said*,* ‘Well*,* maybe you just imagined back then that they were gossiping about you.’ That was not helpful at all in that moment. — BO15*,* Pos. 9*,* about the Police*,* Outreach*.

Others described that the police gave them a good feeling through their polite and kind manner, providing a sense of security that nothing more could happen.

*So the situation itself was perhaps a bit reassuring*,* and I thought*,* ‘Okay*,* now nothing can really happen to me. — BO15*,* Pos. 13*,* about the Police*,* Outreach*.

It was also perceived as particularly calming and helpful when officers attempted to distract the affected person from the situation.

*They […] tried to distract me*,* just by talking about music. To take my mind off things a bit*,* which was also quite good of them in a way. — BO15*,* Pos. 15*,* about the Police*,* Outreach*.

However, in several cases, the behaviour of the police was also described as intrusive and unfriendly, which prevented the development of any trust.

*Because I couldn’t really talk well with the police. I don’t know*,* they weren’t exactly unfriendly*,* but somehow there was no trust at all. I was pretty desperate*,* and they didn’t want to believe me either. — BO3*,* Pos. 3*,* about the Police*,* Outreach*.

One participant reported feeling very unsafe and, as a result, stopped speaking because they felt cornered.

*The police officers were not friendly at all. Totally intrusive*,* […] really cornering me. And I just shut down and didn’t speak at all because I didn’t feel safe with the police there. Exactly. So*,* it wasn’t pleasant. — BO7*,* pos. 7*,* about the Police*,* Outreach*.

It was also described that the police’s behaviour often had an escalating rather than de-escalating effect, both on the situation itself and on the behaviour of the affected person.

*Especially in a situation like that […] someone should have come to de-escalate a bit. Not someone who comes and breaks us up*,* issues tickets*,* throws one person out*,* or bans them from the premises. I don’t know. […] I also think that they were relatively rough. — BO3*,* Pos. 3 + 7*,* about the Police*,* Outreach*.

One participant reported being handcuffed by the police due to their own aggressive behaviour. In retrospect, however, they felt this action was justified to defuse the situation.

*Then I hit the wall*,* and they put handcuffs on me. […] I can also understand why they put them on*,* because they don’t know what kind of situation they’re getting into. They probably did it for self-protection*,* so I wouldn’t hurt myself further. — BO15*,* Pos. 15 + 22*,* about the Police*,* Outreach*.

The same participant further described that, in the situation, they ultimately felt resigned after being overwhelmed and restrained by the police in their own home.

*Yes*,* exactly*,* so really*,* it was already coercion anyway*,* because they had already entered and held me down. But to take the last step*,* where I would really resist completely*,* I just didn’t feel like dealing with that kind of situation anymore. — BO15*,* Pos. 34*,* about the Police*,* Outreach*.

Another participant, who had to wait at the police station for an assessment by the CIS, described feeling trapped in the situation and questioned why they could be forced to wait there.

*I felt locked in. At the moment when they said*,* we’ve called the Social Service and you can’t leave. And I thought*,* I am an individual. I am a human being. Human dignity is inviolable. […] Even if I were suicidal*,* why couldn’t I leave? […] I wasn’t aggressive or anything. I just felt like a second-class human being.* — *BM1*,* Pos. 30 + 39*,* about the Police*,* Outreach*.

## Discussion

This qualitative study explored how individuals experiencing acute mental health crises perceive the care they receive and identified the factors that shape their assessments of care quality. The analysis particularly highlights how service users view the support provided by PSW in acute crisis settings, offering valuable and previously underexamined insights.

### The role of communicative style from the participants’ perspective

Participants described an attentive and empathetic communicative style as central to effective crisis support. Active listening, non‑judgmental behaviour, and emotional validation helped them feel understood and less isolated, reinforcing that their crisis was an illness rather than a personal failing.

These findings align with previous research in various clinical contexts, which has consistently identified empathetic and respectful interaction as a fundamental component of effective therapeutic relationships (24; 31; 26). Participants also emphasised the importance of being informed about potential next steps and engaging in joint reflection on available courses of action. This collaborative approach was experienced as particularly supportive—an aspect similarly highlighted by Mielau et al. [16] in their study on service users’ perspectives regarding the prevention of coercive psychiatric interventions in inpatient settings.

Crises can vary widely in their manifestation, shaped both by individual circumstances and by the dynamics of the specific setting. In acute situations, establishing a rapid and trusting connection between those affected and care providers is essential. Previous research has largely examined this relational dimension from the providers’ perspective. In particular, a recent scoping review by Steimle et al. [24] synthesised existing evidence and underscored the centrality of effective communication and a supportive therapeutic stance in crisis interventions.

Our findings extend this body of work by demonstrating that individuals experiencing acute mental health crises likewise view communication as a crucial foundation for building a supportive relationship. From their perspective, communicative quality is not merely an adjunct to crisis intervention but a core component of feeling safe, understood, and adequately supported.

### Decision-making between autonomy and overwhelm

Participants evaluated decision-making processes positively when providers communicated treatment options transparently, acknowledged individual preferences, and negotiated decisions collaboratively. This strengthened their sense of dignity and self-efficacy.

However, the results of the present study also indicate that shared decision-making in acute crisis situations is not automatically experienced as relieving. Some participants reported feeling overwhelmed during the acute phase because they lacked clear guidance and concrete recommendations. They de-sired more explicit guidance and targeted support in making decisions. Taken together, these accounts suggest the need for a situation-sensitive approach that considers the individuals’ decision-making capacity and balances supportive guidance with the preservation of autonomy. Accordingly, the evidence-based guideline of the Association of the Scientific Medical Societies in Germany (AWMF) [32] emphasizes that shared decision-making requires support tailored to the specific abilities of those affected.

Participants described decisions they experienced as one-sided or imposed—particularly during compulsory admissions involving the police or CIS—as triggering feelings of pressure, powerlessness, and resignation, even when they later considered the measures appropriate. This reflects the ambivalence commonly associated with coercive interventions [33]. Based on these accounts, we infer that it is crucial to make decision-making processes transparent and comprehensible, even when individuals have limited decision-making capacity.

### Structural and personnel-related conditions

At the onset of a crisis, some participants reported feeling misunderstood or perceiving care providers as insufficiently familiar with their individual situation. In several cases, involving an additional professional helped stabilise the situation and strengthened the sense of being supported. This finding points to a broader implication: such interventions, however, require adequate staffing to allow for flexible responses to individual needs. Participants also emphasised that an existing relationship of trust from previous contacts proved particularly helpful, as it facilitated the acceptance of support and contributed to emotional stabilisation.

Our findings show that professional skills play a central role in supporting individuals in acute crises. Participants highlighted two key factors: prior familiarity with staff facilitated trust, and the match between a staff member’s expertise and the individual’s presenting needs proved critical — mismatches undermined the quality of support. These findings align with the scoping review by Steimle et al. [24], who identifiy staff continuity, and prior experience as contributors to high-quality care. In addition, the personal fit between care providers and those affected emerges as a critical factor for successful crisis intervention [24].

Beyond personal factors, the physical environment in which care takes place plays an important role. In outpatient settings, for example, participants expressed a desire for a lounge area that would allow them to withdraw from stressful waiting-room situations. They suggested that a professional should visit this space regularly to provide consistent, low-threshold opportunities for contact.

One approach that addresses these needs is the concept of so-called Safe Spaces. These settings function as accessible points of support for people in crisis and ideally help prevent a direct escalation to the emergency department. In a study conducted by Chakouch et al. [34], several Australian communities implemented peer-led Safe Spaces that offered not only areas for rest and retreat but also active support through conversations with PSW. The findings suggest that using Safe Spaces during acute crises can reduce stress and may represent a promising alternative to emergency department visits [34].

### Crisis follow-up as a gap in care

Regardless of how the intervention unfolded, almost all participants reported that no debriefing of the crisis situation took place. Although guidelines recommend debriefings—particularly in the context of coercion and violence—these practices are still not implemented consistently [35]. Many participants expressed a desire for such conversations, even when they had not experienced coercion.

Peer support appears particularly promising for debriefing and for providing ongoing support after acute mental health crises. Stolz et al. [36] demonstrated positive effects when PSW participated in debriefings following coercive measures. Findings from an additional survey with PSW and CIS staff, conducted as part of the present study, similarly indicate that PSW hold considerable potential for follow-up care and longer-term support aimed at preventing further crises [37].

Beyond the debriefing, such follow-up contact can also provide an opportunity to work with those affected to develop Psychiatric Advance Directives (PADs), which allow individuals to articulate their preferences regarding treatment, communication, and support in the event of a future crisis while in a clinically stable phase [38]. PADs have been shown to increase patients’ sense of autonomy, control, and empowerment [39], and to reduce the risk of compulsory admissions [40]. Drawing on their lived experience and the trust established through previous contacts, PSWs may be particularly well placed to support service users in this process.

### Importance and potential of peer support from the participants’ perspective

In examining the perceived role of peer support in acute, out-of-hospital psychiatric crisis contexts, participants reported particularly positive experiences in outpatient settings. They described PSW as especially empathetic. By sharing their own experiences of crises, PSW conveyed a sense of understanding and reassured participants that they were not alone in their situation.

The New Zealand study by Stainforth et al. [31] reports similar findings. The authors examined the experiences of 20 individuals with suicidal thoughts or attempts, as well as their expectations of crisis teams. Their results show that support provided by “someone who has been through the same experience” and can speak from personal experience was perceived as particularly helpful in acute crisis care. For professional care providers, this often requires balancing professional distance with personal closeness and openness. PSW, by contrast, can step outside the formal professional role and engage with individuals on a different level by drawing on shared lived experience to build trust and establish rapport in ways that differ from traditional clinical interactions, as service users in our study and in previous research have consistently described [31].

Some participants also emphasised that PSW are less constrained by rigid protocols and institutional structures. This flexibility enables them to respond more individually to the needs of the person in crisis, address fears, and highlight possible ways of moving beyond the acute situation. Time availability was identified as another important factor: PSW were perceived as having more time for conversations and support This suggests that, particularly in the context of staffing shortages PSW involvement could help relieve professional staff.

However, the involvement of PSW in situations involving imminent coercive measures was viewed by some participants as potentially risky. This concern was expressed particularly in relation to acute risks of self-harm or harm to others. Similar reservations emerged in focus groups with staff from the CIS in Bremen regarding collaboration with PSW. Staff noted that PSW can be highly valuable in certain situations—for example, when supporting individuals with depression who remain responsive—but may be less helpful or effective for individuals experiencing psychotic states [37].

### Safety versus escalation risk: the role of the police

The previously described positive aspects of respectful and empathetic interaction apply in principle to all actors involved in crisis response—including the police. At the same time, the findings show that problematic experiences were frequently associated with police interventions, underscoring the need for a nuanced perspective. Some participants described polite and de-escalating communication by police officers, which they experienced as protective and calming. Others, however, reported rough or escalating behaviour that heightened fear, mistrust, and withdrawal. These differences may also reflect the fact that police encounters in mental health crises can involve particularly challenging and potentially coercive situations, which may be experienced as more distressing compared to interactions with other service providers.

An Australian study by Roennfeldt et al. [41], which explored the experiences of 31 individuals in mental health crises through qualitative interviews, reported similar patterns. Some participants described empathetic and respectful interactions with police as positive, while others recounted hostile and dehumanising behaviour. Coercive measures that were insufficiently explained were experienced as particularly distressing.

One possible explanation is that police officers often feel uncertain when interacting with people in mental health crises. Wittmann et al. [42], in their study on the effectiveness of a trialogical, contact-based anti-stigma training involving 1,318 young police officers in Germany, found that officers frequently feel inadequately prepared for such situations, and many consider communication with individuals in crisis particularly challenging. Building on this evidence, it can be inferred that targeted training can reduce these uncertainties, encourage reflection on stereotypical assumptions, and strengthen skills in empathetic and de-escalating communication, thereby supporting non-violent interventions [42].

It should be noted that several factors mentioned in this discussion — including the physical layout of the waiting area, the involvement of the police, and the conditions under which PSW were integrated — varied considerably between outreach and outpatient settings. Although the present study was not designed to compare these settings, and the sample size does not allow for definitive conclusions, these differences may meaningfully shape participants’ experiences and warrant closer examination in future research.

### Strengths and limitations

A central strength of the study is that it successfully included vulnerable individuals who are often difficult to reach for research due to their mental health conditions or stressful life situations. Especially in highly dynamic and relationship-dependent contexts, this perspective provides important insights into how professional interventions are perceived and which factors play a role. This is particularly significant because the benefit of support does not depend solely on what is objectively provided, but largely on how it is experienced and evaluated by those affected [25].

Different settings and intervention formats were included, providing a comprehensive overview of the diversity of care situations in practice. The study provided outpatient and outreach crisis interventions, with and without police involvement. For the first time, PSW participation in a non-clinical acute psychiatric setting was investigated. This nuanced presentation of perspectives enhances the practical relevance of the findings.

At the same time, several limitations should be considered. While the final sample was slightly smaller than planned, the interviews provided rich and meaningful insights and allowed us to approach thematic saturation in several subgroups, even though some groups were more difficult to include. The interviews were conducted, on average, about three months after the crisis interventions. This timing represented a compromise: on the one hand, enough time needed to pass to avoid placing additional burden on participants; on the other hand, the delay could not be too long so that important details would not be forgotten. As with all qualitative interview-based research, the findings reflect participants’ personal perspectives and interpretations of their experiences, which may be shaped by recall bias or the time elapsed since the crisis intervention. Furthermore, perceptions — particularly during acute psychological crises — are strongly influenced by individual emotional states, fear, and disorientation, and interactions are reciprocal, meaning that the behaviour of professionals directly responds to that of those affected. These aspects are inherent to the nature of subjective experience and do not diminish the validity of participants’ accounts, but should be considered when interpreting the findings.

Another limitation is that participants without prior experience with PSW were introduced to the peer support concept during the interview. Consequently their assessments were based on the information provided within the interview context rather than on personal experience. As a result, their responses may have been influenced by the manner in which the concept was explained.

Self-selection effects are also possible, as individuals with particularly intense experiences—both positive and negative—may have been more willing to participate. In addition, only individuals who felt capable of taking part in such an interview may have participated, which suggests that the sample may not fully represent all eligible individuals.

## Conclusions

Our research examined service users’ perspectives on acute, outreach, and outpatient psychiatric crisis interventions in routine practice. A distinctive feature of this study is its focus on peer support workers embedded in psychiatric crisis services—an approach that remains relatively novel in acute non-inpatient mental health care.

From the perspective of participants, interactions perceived as calm, attentive, and empathetic were experienced as supportive for feelings of safety and being understood. In particular, a respectful, non-judgmental attitude from care providers, as well as clear and transparent communication, helped build trust and promote emotional stabilization. In contrast, interactions experienced as intrusive, insensitive, or insufficiently explanatory were often perceived as a lack of compassion and were associated with feelings of being misunderstood, uncertainty, or withdrawal.

Positive effects of respectful and empathetic interactions were reported for all types of care providers, including the police. At the same time, problematic experiences were reported more frequently with police than with other providers, highlighting the need for a nuanced interpretation. This may partly reflect the particularly challenging and at times potentially coercive nature of police involvement in mental health crises, which can be perceived as more distressing. To reduce negative experiences and prevent escalation, targeted training to strengthen communication and de-escalation skills in interactions with people in mental health crises appears to be essential.

From the participants’ perspective, PSW may have a valuable complementary role in this context. However, it should be noted that only a small number of participants had direct experience of peer support; the majority expressed views on its perceived potential value rather than reporting first-hand experiences. With this caveat in mind, participants suggested that PSW, by drawing on their own crisis experiences, can convey a unique form of empathy and credibility. This could reduce fear, lessen feelings of isolation, and foster hope by not only discussing recovery but also embodying it. At the same time, the findings suggest that peer support should be applied selectively and may not be equally suitable in all acute situations. Against this background, further research is needed to examine the specific mechanisms, applications, and limitations of peer support in acute crisis situations in more detail and to continue developing evidence-based approaches. Beyond the acute phase, peer-facilitated follow-up — for instance through the development of PADs — may represent a promising avenue to support service users in preparing for future crises and reducing the risk of compulsory admissions. This points to the potential value of integrating PSW more systematically into structured follow-up care, rather than limiting their involvement to the acute crisis phase.

## Supplementary Information

Below is the link to the electronic supplementary material.

Supplementary Material 1

## Data Availability

The interview guide is included in the appendix of this article. The coding framework is available from the authors upon reasonable request. The underlying data, in particular the full interview transcripts, are not publicly available due to participant anonymity and confidentiality constraints.

## Abbreviations

CIS: Crisis Intervention Services
Cluster-RCT: Cluster-randomized controlled trial
DRKS: German Clinical Trials Register
EmPeeRie NoW: Trialogical advisory board of the Department of Psychiatry and Psychotherapy at the University Medical Centre Hamburg-Eppendorf
EX-IN: Experienced Involvement
GeNo: Gesundheit Nord
ISCED: International Standard Classification of Education
PSW: Peer Support Worker(s)
