# Supplementary material for: Service users’ perspectives on acute, outreach and outpatient psychiatric crisis interventions with and without peer support: results of a qualitative study
Source: BMC Health Serv Res. 2026 Aug 11;26:1140. doi: 10.1186/s12913-026-15319-y (PMC13479813; doi:10.1186/s12913-026-15319-y)
Supplement: Supplementary file 1 [file 12913_2026_15319_MOESM1_ESM.pdf]

Interviewguide_Peer Intervent_Patients

Interview Section	Guiding Question	Follow-Up Questions / Memo
1 Introduction	Did you find your way here all right? Would you like something to drink? How are you?<br><br>You know why you're here. Is there anything you need to feel comfortable and able to talk about the topic?	Thank you for participating in the interview. I will introduce myself… talk about privacy, provide information about the interview, emphasize voluntariness, obtain informed consent, offer an opportunity for questions, mention the interview's goal: improving care by incorporating the perspective of those affected and enhancing their quality of life; inquire about needs and clarify that breaks are possible at any time; This interview is about your personal experiences, so there are no right or wrong answers; I'll be taking notes occasionally, please don't be alarmed; and I'll announce the start of the audio recording.
2 Crises Experiences	a) Can you please tell me about the day you visited the Social Psychiatric Counseling Center / had contact with the staff? If you feel comfortable, we can also talk about any previous contact you may have had with the Social Psychiatric Service before that.<br><br>b) You've just described how the day went until the contact with the Social Psychiatric Service. Now, thinking back to that: How did it go? Please tell me more about your experience!	- What happened on that day before you contacted the Social Psychiatric Service?<br>- Was anything different than usual?<br>- How were you feeling that day?<br>- What triggered the situation that led to this contact?<br>- Do you have an idea how the situation came about?<br><br>- What were you hoping for from the conversation at the social psychiatric counseling center?<br>- How did you experience/feel about the situation? (What were the positive aspects, what were the negative aspects?)<br>- Who was present in the situation? (Police, crisis intervention team, family members...) Who was there first, and who arrived later?<br>- Did the people present introduce themselves? If so, how did they introduce themselves?<br>- What did the people present do?

		- Was the role of the Peer Support Worker explained?<br>- Was the Peer Support Worker introduced?<br>- Did you notice the presence of a Peer Support Worker? How did you notice it?<br>- Did you understand the role of the Peer Support Worker?<br>- How did the Peer Support Worker initiate contact?<br>- How did you feel about the presence of the Peer Support Worker?<br>- Did you feel acknowledged?<br>- Were the people present helpful to you?<br>- How did the support feel?<br>- What support would you have wished for? What would have helped you?<br>- How did this treatment feel for you? Could you please describe how you would have preferred to be treated?<br>*Now we would like to move on to some more challenging questions about the course of the intervention. Are you ready for that?*<br>- Did you feel pressured? Did you feel threatened? Did you experience threats of coercion?<br>- Were there coercive measures? (Involuntary commitment, restraint, informal coercion...)<br>- What kind of coercion was applied? How exactly did this happen? Who was involved?<br>- Was the presence of the Peer Support Worker helpful in the situation? Did the Peer Support Worker support you?<br>- From your current perspective, what would you have needed to avoid coercion?
	c) What happened after that contact?	- What did you do after the intervention? Where did you go?<br>- Was the intervention discussed with you afterwards?<br>- What impact did the intervention have on your health/recovery?<br>- Were there any personal changes after the intervention? Are there things you see differently since then? If yes, what are they?

	d) I would appreciate it if you could tell me about your previous experiences with crises, if you feel comfortable doing so.	- What may have been different compared to the crisis three months ago?<br>- In what ways was the situation better or different, and how did you manage to resolve it?
3 Closing	“We have now reached the end from my side. Is there anything else that is important to you and that we have not yet discussed in our conversation?”	
